# Impact of Functionalized Graphene Nanoplatelet Incorporation on the Properties of 3D-Printed Resin-Based Dental Composites

**DOI:** 10.1021/acsomega.6c04462

**Published:** 2026-06-29

**Authors:** Lilian Vanessa Rossa Beltrami, Maria Eduarda Pereira Goulart, Rafaele Frassini, Mariana Roesch Ely, Thiago de Oliveira Gamba, Diego Piazza, Ademir José Zattera

**Affiliations:** † Postgraduate Program in Process and Technology Engineering (PGEPROTEC), 58802 University of Caxias do Sul (UCS), CEP 95070-560 Caxias do Sul, RS, Brazil; ‡ Laboratory of Applied Toxicology and Bioproducts, University of Caxias do Sul (UCS), CEP 95070-560 Caxias do Sul, RS, Brazil; § Department of Surgery and Orthopedics, Dental School, Federal University of Rio Grande do Sul (UFRGS), CEP 90035-003 Porto Alegre, Brazil

## Abstract

This study aims to evaluate the impact of graphene nanoplatelets functionalized with different silanes on the mechanical, thermal, and morphological properties of a resin, as well as to determine how these modifications influence the overall structural performance of the material when applied in dentistry. To prepare the composites, functionalized graphene nanoplatelets were incorporated into photosensitive acrylic resin at a concentration of 0.250% (w/w) by using the sonication method. Subsequently, the samples were fabricated through 3D printing. Characterization of the composites was performed through Raman spectroscopy, contact angle measurements, abrasion resistance analysis, thermomechanical analysis, and cytotoxicity testing. The incorporation of silane-functionalized nanoplatelets resulted in enhanced chemical modification of the resin surface, rendering the composite hydrophobic and reducing mass loss due to abrasion, particularly when 3-(trimethoxysilyl)­propyl methacrylate (TMPSP)-functionalized nanoplatelets were used. However, greater material deformation and expansion were observed when incorporating pure or silanized graphene nanoplatelets. Cytotoxicity testing demonstrated that the materials utilized in this study do not induce adverse health reactions over a specific period. With significant advancements in 3D printing technology and the growing market for nanotechnology and nanomaterials, various sectors, including dentistry and medicine, have undergone transformative changes, leading to improvements in treatment protocols and healthcare practices. The findings of this study are particularly important, as they pave the way for the development of high-performance dental composites with tailored properties for specific applications. The successful incorporation of functionalized graphene nanoplatelets into resin-based composites offers a promising strategy to enhance critical factors for the durability and longevity of dental restorations. Moreover, the use of 3D printing technology provides precision and customization, enabling the production of complex geometries with superior structural integrity. Thus, the integration of nanotechnology and additive manufacturing represents a significant step toward the development of innovative dental materials capable of meeting increasing expectations for biocompatibility, functionality, and long-term stability.

## Graphical abstract

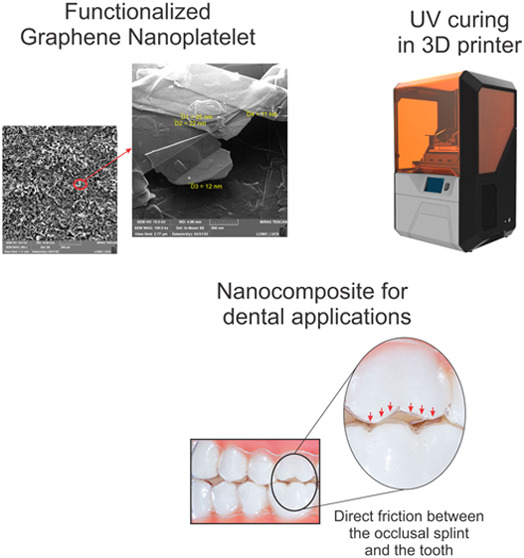

## 1 Introduction

The advent of additive manufacturing technology, commonly termed three-dimensional (3D) printing, has brought forth technological advancements across various domains. This technology enables the layer-by-layer fabrication of 3D objects through computer-aided design (CAD).^1,2^ In dentistry, this technology has become routinely used in various applications, encompassing the production of dental models, surgical and orthodontic guides, bruxism splints, fixed or removable dental prostheses, custom trays, dental implants, and more.^3–5^

Printable materials, primarily photosensitive resins composed of a combination of multifunctional monomers based on (meth)­acrylate photoinitiators, additives, and diluents, are commonly employed to produce these 3D structures.^6–8^ Numerous 3D printers employing additive manufacturing processes, such as stereolithography (SLA) and digital light processing, are commercially available.^9^ Unlike traditional production methods, these SLA/DLP printing technologies require only a CAD model containing all of the information about the part to be printed, eliminating the need for molds.^10^ These technologies offer benefits such as time and cost savings, enabling swift adaptation to address the evolving needs of the industry.^10^

The SLA/DLP printing technologies rely on a light source, whether ultraviolet (UV) lasers or UV LED lights, to polymerize liquid polymers, which solidifies the resin layer by layer, resulting in 3D structures.^9^ The accuracy of printed objects can be influenced by several factors, including the 3D printer model, printing parameters, materials used, and the manufacturing process itself.^11,12^ Moreover, manufacturers of photosensitive resins designed for SLA/DLP printing advise postprocessing treatment, known as postcuring, involving either thermal or UV light curing. This step facilitates the reaction of nonreticulated monomers to complete the polymerization process after printing, transforming the material from a viscous state to a rigid one.^5^ The postpolymerization process plays a pivotal role in the thermal and mechanical properties of the material.^5,13^

The commercial popularization of carbon-based nanofillers, notably graphene, a two-dimensional (2D) carbon-based nanomaterial, heralded as the thinnest and strongest known material,^14,15^ has enabled the development of lighter and more resistant nanocomposites suitable for a wide array of applications.^16^

Nanocomposites can be considered nanomaterials when composed of two or more distinct chemical compositions or forms,^17^ encompassing fibers, particles, or 2D materials embedded within a polymer matrix,^18^ as well as possessing a dispersed phase on a nanometric scale in at least one of their dimensions.^19^ The manufacturing of graphene- and resin-based nanocomposites using 3D printing has enabled the development of devices with enhanced precision and improved mechanical, electrical, and thermal properties.^20^ Recent investigations have examined the effects of nanoparticle addition within the polymeric matrices of resins, revealing the occurrence of material integration and nanoparticle-mediated interfacial improvements that enhance layer-to-layer connectivity.^21,22^

According to Lorusso et al., graphene stands as an ideal nanomaterial for enhancing the performance of acrylic resins in dental applications. Notably, the authors evaluated the properties of PMMA with added graphene for the production of dental implants.^23^ Similarly, Lopez de Armentia et al. explored the incorporation of graphene and its derivatives into a commercial photosensitive resin, investigating their impacts on thermal, rheological, and physicochemical properties, dimensional accuracy, 3D printability, and polymerization reaction.^24^

In the current work, SLA printing technology was employed to produce nanocomposites based on urethane methacrylate resin augmented with graphene nanoplatelets treated with different types of silanes. Furthermore, this study aimed to evaluate the influence of this additive on the mechanical, thermal, and cytotoxicity properties of the resin to demonstrate its relationship with the broader structural performance when applied in the field of dentistry.

## 2 Materials and Methods

### 2.1 Materials

In this study, the photosensitive acrylic resin Smart Print Bio Bite Split (Smart Dent, São Carlos, São Paulo, Brazil), which is curable by UVA/UVB 405 nm light, was used. According to supplier specifications, this resin is specifically designed for fabricating interocclusal devices using 3D printing. It boasts a viscosity of 0.5 Pa·s and a density of 1.2 g/cm^3^.

Graphene nanoplatelets (GNPs) were sourced from UCSGraphene, grade UGZ-1001, with distinctive properties including a specific superficial area (BET) of 11.25 m^2^/g and a carbon content exceeding 93% by mass. For the chemical modification of the GNP surface, the following alkoxide precursors (silanes) were used: 3-(trimethoxysilyl)­propyl methacrylate (TMSPM), 3-aminopropyltriethoxysilate (APTES), and 3-glycidoxypropyltrimethoxysilane (GPTMS), all obtained from Sigma-Aldrich.

### 2.2 Methods

#### 2.2.1 Surface Chemical Modification of GNP

Initially, GNP was dispersed in 50 mL of ethanol through sonication using VibraCell equipment for 1 h, in two cycles of 30 min, with a 5 min interval between each, carried out in an ice bath. The sonication was performed at an amplitude of 40% and a nominal power of the equipment at 500 W.

For the alcoholysis of silanes, a 100 mL solution of ethanol/distilled water in a 75:25 (v/v) ratio was prepared. Subsequently, the silane (TMSPM, APTES, or GPTMS) was added at a ratio of 3 mL per 1 g of GNP to be modified. The solution was kept under magnetic stirring for 1 h at room temperature. Then, the previously prepared ethanol/GNP suspension was incorporated into the specific silane solution and continuously stirred for 4 h at 70 °C. Following this, the suspension was centrifuged and washed (75:25 ethanol/distilled water) to eliminate any unreacted silanol groups. Lastly, the suspension was dried in an 80 °C oven for 12 h. The aforementioned GNP surface chemical modification process was based on literature references.^25,26^

#### 2.2.2 Composite Production

To produce the composites, GNP (with or without surface chemical modification) was incorporated into the photosensitive acrylic resin at a concentration of 0.250% (w/w) using the sonication method with an amplitude of 40% for 30 min in an ice bath. Subsequently, the composite samples were produced using a rapid prototyping Flashforge 3D printer (dOne 3D, Ribeirão Preto, São Paulo, Brazil), employing digital light processing (DLP) stereolithography technology (Figure 1a). To design the sample bodies, the 3D DLP FlashDLPrint accompanying software was used, and the files were saved in STL (Standard Triangle Language) format. Each part was crafted according to the planned tests, and the printing orientation was set to vertical. The printing standards, as recommended by the manufacturer, are 3.5 s for curing time, transition layers of 8, 20 s for adhesion layers, and 40% of the light intensity. It should be noted that the printing parameters provided by the resin manufacturer were not modified for the nanocomposites, and there were no changes to the final printing results. Following printing, the samples were immersed in analytical grade isopropyl alcohol for 10 min, and subsequently subjected to a postcuring process for 5 min in a curing oven (dOne 3D, Ribeirão Preto, São Paulo, Brazil) under 405 nm UV light and a power output of 40 W, following the instructions provided by the acrylic resin supplier. The images of the samples prepared for the Abrasion Resistance and Thermomechanical Analysis (TMA) tests, as well as their dimensions, are shown in Figure 1b,c, respectively.

**Figure 1:**
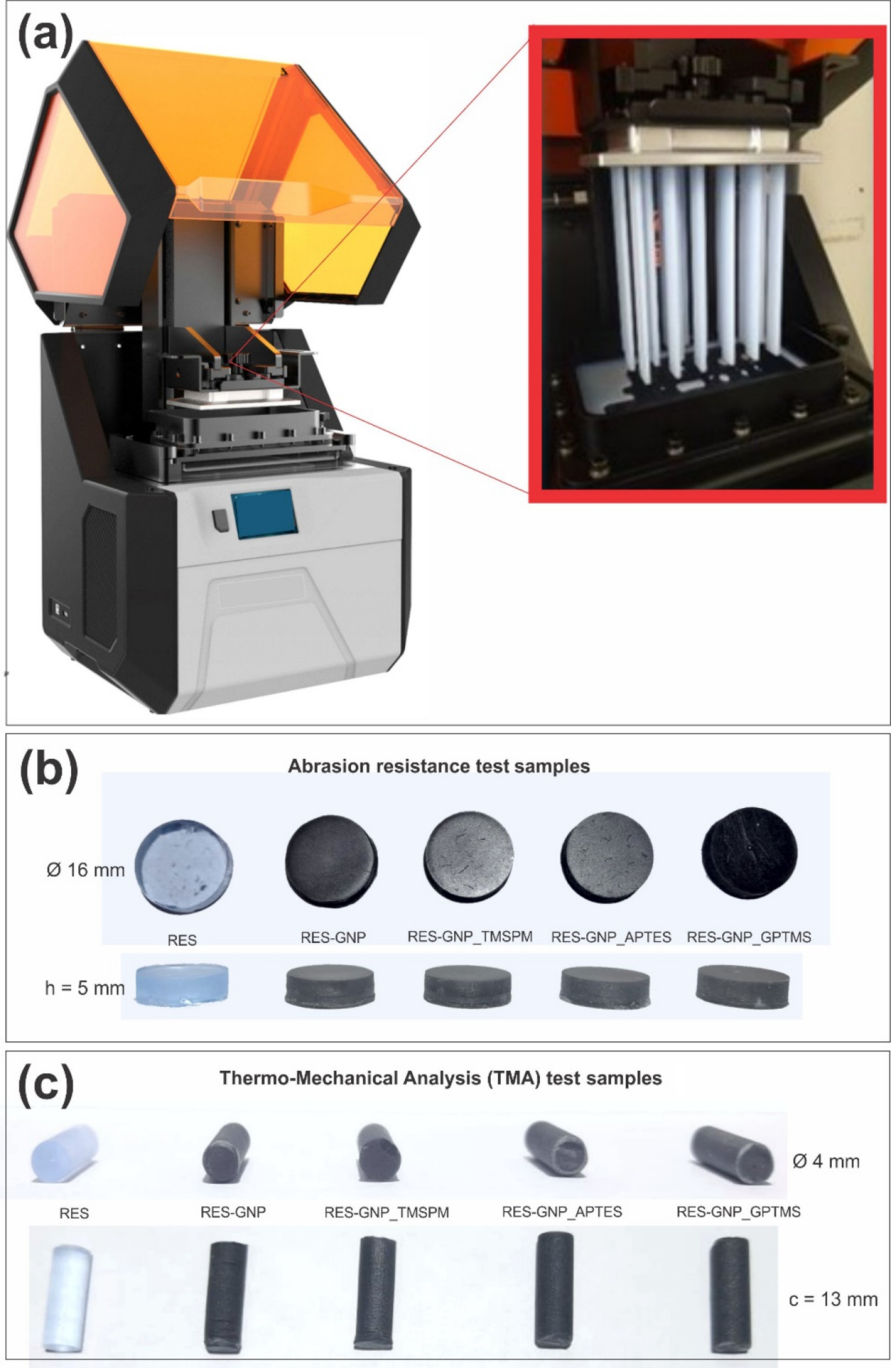
Photographs of the rapid prototyping Flashforge 3D printer (a) and samples prepared for the: (b) abrasion resistance and (c) thermomechanical analysis (TMA) tests, as well as their dimensions.

#### 2.2.3 Characterization

The surface chemical modification of the GNP was assessed by using Raman spectroscopy. This analysis method was conducted using a Renishaw inVia Raman spectrometer, equipped with a 532 nm laser set at 5% and a 50× magnification lens.

Wettability of the composites was determined through static contact angle measurements using the sessile drop method. In this procedure, a drop of deionized water was deposited on the sample using a B-D Yale 3D syringe, photographed, and the image was analyzed using the Surftens 3.0 software to measure the resulting angle. All measurements were performed in triplicate, with three droplets placed on different points of the surface. The test was conducted at a constant temperature of 23 °C, using a droplet volume of 10 μL.

The abrasion resistance was assessed by measuring the progressive mass loss of the composites when subjected to mechanical wear via grinding. The assay was performed in triplicate according to ASTM D5963-04 and employed a MaqTest abrasion tester equipped with a standard grit size 60 sandpaper and an abrasion path length of 40 m. Sample mass loss was determined using a high-precision balance.

Thermo-mechanical analysis (TMA) of the composites was performed following ASTM E831-14 standards, using specimens measuring 8 mm × 8 mm × 4 mm. The tests were carried out using a Shimadzu TMA-60 instrument, employing a temperature ramp from 30 to 170 °C at a rate of 5 °C/min, with a 5 N load cell.

The cytotoxicity of the samples was evaluated in vitro according to the ISO 10993-5 standard (elution test method). Sample fluid extracts were diluted in Dulbecco’s Modified Eagle Medium (DMEM) supplemented with 10% v/v fetal bovine serum and 1% penicillin/streptomycin (100 units/mL). Mouse fibroblast cells (L929) were plated at a concentration of 5 × 104 cells·mL^–1^ in a 96-well plate and incubated in an oven at 37 °C in an atmosphere of 5% CO_2_. The samples were treated with the extraction solutions for a period of 1, 2, and 7 days. After the period of each treatment, the culture medium was removed and the MTT solution [3-(4,5-dimethylthiazol-2-yl)-2,5-diphenyltetrazolium bromide] (0.4 mg·mL^–1^) by 2 h. Then, the MTT solution was removed, and the formation crystals were solubilized in DMSO. The optical density (OD) was determined by reading in a spectrophotometer (Max190 spectra, Molecular Devices, USA) at 570 nm. Cell viability (%) was calculated by the OD of the treated group/OD of the control group × 100. The results represent the average of three different experiments performed in triplicate.

## 3 Results

### 3.1 Raman Spectroscopy

Figure 2 illustrates the Raman spectroscopy results for graphene nanoplatelet samples with or without functionalization. This analysis serves to identify, assess the quality, and evaluate the structural performance of graphene nanoplatelets based on the intensity, frequency, and line width of their G, D, and 2D modes.

**Figure 2:**
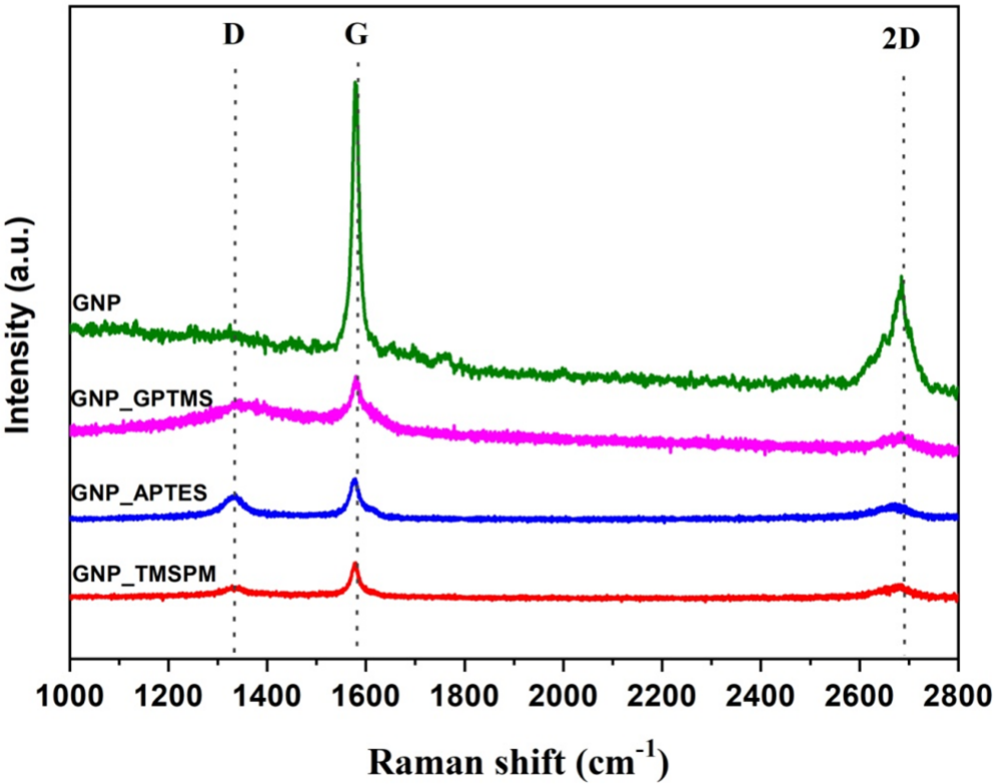
Raman spectra obtained for the graphene nanoplatelet samples with and without functionalization.

The Raman spectra exhibit characteristic bands of carbonaceous materials. The presence of the G band at approximately 1582 cm^–1^ is related to the in-plane sp^2^ vibration of C atoms.^27^ The 2D band, at 2721 cm^–1^, predicts the number of graphene layers.^28–30^ The D band, often termed the disorder band, corresponds to the presence of vacancies or distortions in carbon rings and arises due to the lattice movement away from the Brillouin zone center, and its presence is noted between 1270 and 1450 cm^–1^.^30^ The absence of the D band in the GNP samples indicates the nonexistence of functional groups on the surface or few defects in the hexagonal lattice of C atoms.^28^

According to Nguyen et al., an *I*_2D_/*I*_G_ ratio of 3 corresponds to single-layer graphene, 2 > *I*_2D_/*I*_G_ > 1 indicates bilayer graphene, and *I*_2D_/*I*_G_ < 1 signifies multilayer graphene. As observed in Table 1, all samples correspond to multilayer graphene. Furthermore, the *I*_D_/*I*_G_ ratio is an important parameter for evaluating the level of disorder within the crystalline structure of a sample. Notably, Table 1 shows its expressive increase for silane-functionalized GNP samples, indicating efficient surface functionalization.^31^

**Table 1:** Intensity Ratio of the Peaks Observed by Raman Spectroscopy for the Graphene Nanoplatelet Samples With and Without Functionalization

sample	intensity of Raman peaks	
*I*_2D_/*I*_G_	*I*_D_/*I*_G_	
GNP	0.39	0.04
GNP_GPTMS	0.33	0.60
GNP_APTES	0.38	0.61
GNP_TMSPM	0.38	0.33

### 3.2 Contact Angle

Figure 3 depicts the contact angle results for the studied samples. Notably, the surface of the pure resin exhibits hydrophilic characteristics, with a contact angle of approximately 75°. This aligns with a study conducted by Huang et al., where the contact angle of pure resin was 72.05°, indicative of it being a hydrophilic material.^30^ The addition of GNP alters the surface energy, rendering the surface of the composite hydrophobic, resulting in a contact angle of approximately 85°. Upon the addition of TMSPM-functionalized GNP, the surface energy of the composite underwent a significant alteration, becoming hydrophobic with a contact angle of approximately 95°.

**Figure 3:**
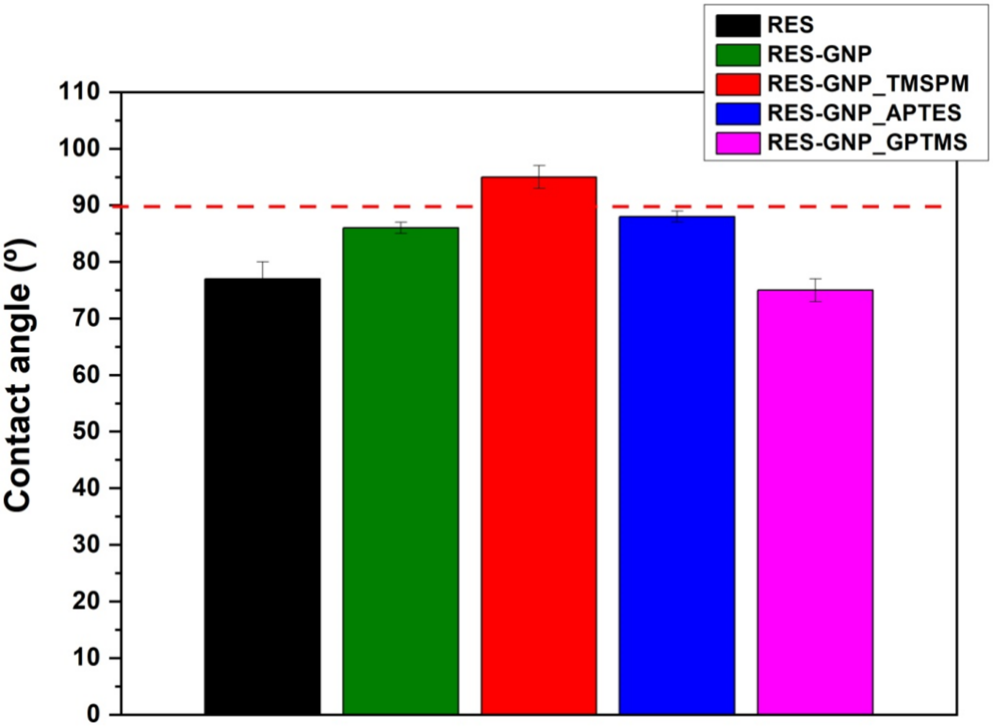
Contact angles obtained for the studied composite samples.

### 3.3 Abrasion Resistance

Figure 4 depicts the outcome of the abrasion resistance assay for the investigated samples. It is known that a coating with low resistance to wear leads to diminished operational durability of the material.^32^ The observed mass loss after the abrasion test for the pure resin and resin containing nonfunctionalized GNP showed minimal alteration, indicating that the addition of GNP does not significantly impact this property. However, the addition of TMSPM-functionalized GNPs leads to a 15% reduction in mass loss after the abrasion test. However, the opposite effect was observed for samples containing GNPs functionalized with APTES and GPTMS silanes. In these cases, the mass loss after abrasion increased by 60% and 20%, respectively.

**Figure 4:**
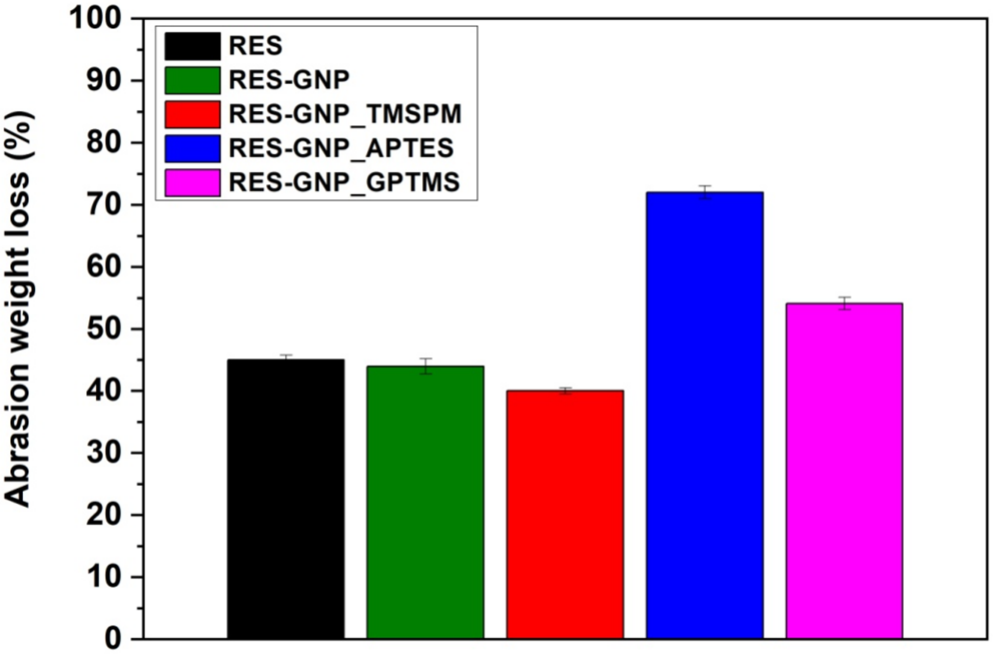
Abrasion resistance obtained for the studied composite samples.

### 3.4 Thermo-Mechanical Analysis

The dimensional stability was explored via TMA (Figure 5). The GNP-containing samples exhibited a slightly higher percentage of deformation compared with the pure resin sample.

**Figure 5:**
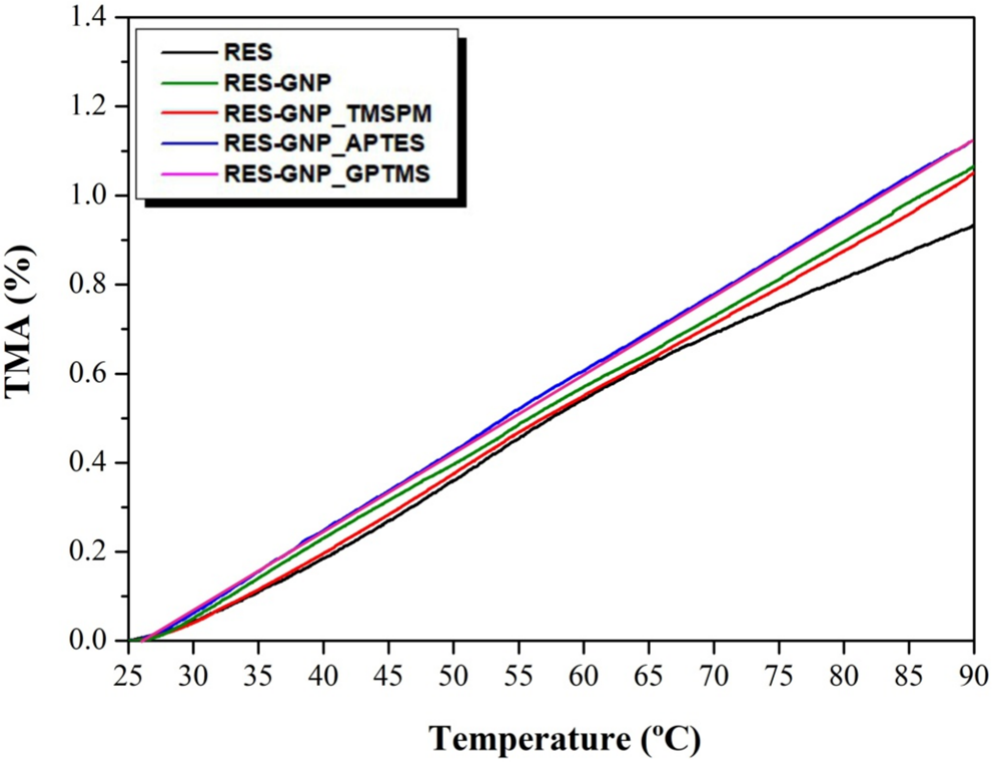
TMA results obtained for the studied composite samples.

The thermal expansion coefficient (α) of the composites was calculated based on the slope of the thermal expansion curves, and the results are presented in Table 2. Two temperature ranges were evaluated: simulating intraoral temperature (25–40 °C) and for industrial applications (40–90 °C). It is observed that the addition of GNP (RES-GNP) increased α compared to the pure resin (RES) within the first evaluated temperature range (25–40 °C).

**Table 2:** Linear Coefficient of Thermal Expansion (α) in the Temperature Ranges of the Studied Composites

sample	α (10^–4^·°C^1–^)	
25–40 °C	40–90 °C	
RES	1.28	1.51
RES-GNP	1.50	1.66
RES-GNP_TMSPM	1.31	1.68
RES-GNP_APTES	1.65	1.75
RES-GNP_GPTMS	1.66	1.75

### 3.5 Cell Viability Assay

Fibroblasts were cultured on 3D printed specimens, and viability assays conducted at 24, 48, and 168 h revealed varying results, as depicted in Figure 6. The highest cell viability was exhibited by the pure resin samples (109%) after a 168-h incubation period. In comparison, the resin containing TMSPM-functionalized GNP (95%) and nonfunctionalized GNP (78%) yielded lower results. When compared within a 48 h interval, higher cell viability was observed in the resin containing TMSPM-functionalized GNP (88%), while other specimens exhibited very similar results (78%). Within a 24-h incubation period, pure resin (97%) and resin containing TMSPM-functionalized GNP (94%) showed almost identical results, whereas resin containing nonfunctionalized GNP (83%) displayed inferior results.

**Figure 6:**
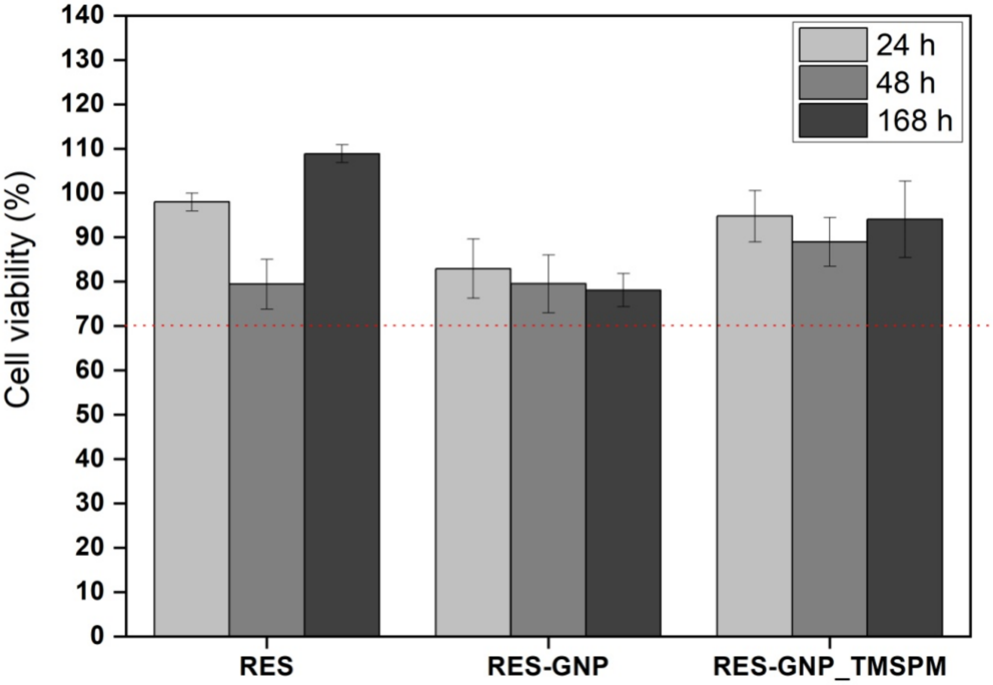
Results of the cell viability assay obtained for the composite samples studied.

## 4 Discussion

The higher intensity of the D-band observed for the GNP_APTES sample in the Raman analysis indicates an increased degree of structural disorder. This behavior can be attributed to the functionalization with APTES, which introduces amine-containing groups on the graphene nanoplatelet surface. Such a modification promotes disruption of the sp^2^ carbon network and the formation of sp^3^-like defects, enhancing the defect-activated D band. Compared to other silane agents, APTES may interact more strongly with the carbon surface due to the presence of reactive amine groups, leading to a more pronounced increase in the defect density.

The results of the contact angle test showed that the surface of the pure resin exhibits hydrophilic characteristics (75°), while the addition of GNP alters the surface energy, giving the surface of the composite an almost hydrophobic character (85°), and the addition of TMSPM-functionalized GNP creates a hydrophobic surface (95°). This result is important concerning the application of these composites, as hydrophobic surfaces typically exhibit reduced biofilm formation. Due to chemical differences between the employed silanes, the results found for the APTES- and GPTMS-functionalized GNP samples did not manifest the same degree of alteration in comparison to the TMSPM-treated sample.

The observed mass loss after the abrasion test for the pure resin and resin containing nonfunctionalized GNP showed minimal alteration. However, the addition of TMSPM-functionalized GNPs decreased the mass loss after the abrasion test, suggesting that TMSPM-functionalized GNP fostered a heightened interaction between the matrix and nanofillers, thus decreasing wear. This improved abrasion resistance is consistent with the enhanced interfacial interaction suggested by increased hydrophobicity. The capacity to withstand the removal of material from a surface due to friction, scraping, or erosion is defined as the resistance to abrasion. Consequently, a coating with low resistance to wear leads to diminished operational durability of the material.^32^

The samples containing GNP functionalized with APTES and GPTMS exhibited higher mass loss after abrasion compared to both the pure resin and the TMSPM-functionalized system. This behavior suggests a less effective interfacial interaction between the nanofillers and the polymer matrix. In the case of APTES, the presence of amine groups may promote strong but localized interactions with the graphene surface, increasing structural defects—as also indicated by Raman analysis—and leading to stress concentration points that facilitate filler pull-out during abrasion. This results in a significant increase in material removal. For GPTMS-functionalized GNP, the intermediate mass loss can be associated with the epoxy functional group, which may not fully react with the polymer matrix under the employed processing conditions, leading to suboptimal interfacial bonding.

Important factors to consider are that the temperature fluctuations in the oral cavity directly affect the dimensional stability of materials, a parameter of extreme relevance for their application in dental settings. The GNP-containing samples exhibited a slightly higher percentage of deformation compared with the pure resin sample. According to Wu et al., higher bonding energies result in less material expansion when subjected to temperature variation. Thus, it can be inferred that the presence of GNP promoted a change in the bonding energy of the polymeric chain, and as the temperature rises, a relaxation of the polymeric chain occurs, leading to material expansion.^33^

With increasing temperature, free chain segments start to move and dissipate excess energy in the form of heat. This temperature elevation is observed in samples with added GNP, suggesting that these composites possess a higher density of cross-linkages, constraining chain mobility.^34^ The thermal expansion coefficient (α) of the composites was calculated based on the slope of the thermal expansion curves, and a low α value is often desired to achieve dimensional stability.^35^

In this context, the addition of GNP (RES-GNP) increased α compared to the pure resin (RES) within the first evaluated temperature range. Hossain, Hossain, Dewan, Hosur, and Jeelani explain that a α within the glass region is related to polymer chain expansion and free volume expansion phenomena, leading to variability in these values.^36^

All samples evaluated showed cell viability results of greater than 70% in all incubation periods. The highest cell viability was exhibited by the pure resin sample, followed by the sample containing TMSPM-functionalized GNP, after a 168-h incubation period. Fibroblasts are the major group of cells present in the gingival connective tissues, performing several functions, being essential in wound healing, as well as producing extracellular matrix.^37,38^ Aati et al. reported that the presence of up to 0.25% by weight of GNP demonstrated a biocompatible response with oral fibroblasts, associated with an insignificant reduction in cell viability.^39^ Graphene-based materials have been linked to potential cytotoxic effects due to the presence of oxygen functional groups, leading to an excess of reactive oxygen species.^40^ It is worth noting that GNP exhibits the lowest oxygen percentage among various types of graphene.^41^

Bayarsaikhan et al. described the association between cytotoxicity and the degree of polymerization on the surface of the material, resulting from both the polymerization during the 3D printing process and the polymerization during postcuring.^5^ In 3D printing technology, there is no chemical reaction process involved, suggesting a potentially lower cytotoxicity compared with conventional production methods using self-polymerizing resins, which entail the occurrence of chemical reactions.^42^

## 5 Conclusion

This study investigated the influence of graphene nanoplatelets treated with different types of silane on the mechanical, thermal, and morphological properties of resin. By this study, the following conclusions can be drawn:

(1) The chemical modification on the surface of the resins in samples with silane-functionalized GNP increased substantially, indicating efficient surface functionalization.
(2) The composites became hydrophobic upon the addition of TMSPM-functionalized GNP, which tends to reduce biofilm formation compared to hydrophilic surfaces.
(3) TMSPM-functionalized GNP samples exhibited a reduced mass loss due to abrasion. Conversely, APTES- and GPTMS-functionalized GNP samples exhibited greater loss of mass due to abrasion, indicating inefficient action of these silanes.
(4) In the thermomechanical analysis, the addition of pure or silanized GNP resulted in slight deformation and expansion of the materials compared to the pure sample.
(5) The present study indicated that the investigated materials did not exhibit cytotoxic effects over the periods of 24, 48, and 168 h.

Among the evaluated systems, TMSPM-functionalized GNP demonstrated the most promising overall performance, combining improved wear resistance and hydrophobicity without compromising biocompatibility. Thus, composites with added TMSPM-functionalized graphene nanoplatelets appear to be promising candidates for various applications in the field of dentistry in the near future. However, further studies are necessary to confirm the biocompatibility of nanomaterials. Moreover, we suggest conducting in vivo studies and bactericidal tests, considering that the oral environment is conducive to the adhesion of various bacteria and microorganisms.
